# Editorial: Cell-mediated innate immunity in aquatic invertebrates and fish: what’s new?

**DOI:** 10.3389/fimmu.2025.1749877

**Published:** 2026-01-07

**Authors:** Maria Rosaria Coscia, Rita Marino, Daniela Melillo

**Affiliations:** 1 Institute of Biochemistry and Cell Biology, National Research Council of Italy, Naples, Italy; 2 Biology and Evolution of Marine Organisms (BEOM), Stazione Zoologica “Anton Dohrn”, Naples, Italy

**Keywords:** innate immune cells, aquatic environment, host-pathogen interaction, aquaculture health management, novel cell functions, pathogen sensing, evolution of innate immunity

The study of defense strategies and innate immune mechanisms in marine organisms—spanning invertebrates and vertebrates—continues to reveal remarkable complexity shaped by various biotic and abiotic pressures in aquatic environments. Far from being a simple first line of defense, innate immunity in these organisms involves sophisticated cellular and molecular processes that rival those of higher vertebrates.

As highlighted in this Research Topic, the innate immune response—including cell-mediated processes—is essential for rapid and effective protection against a broad spectrum of invaders. Recent findings have uncovered surprising immunological diversity and functional similarities among immune cells of invertebrates, fish, and mammals, identifying conserved chemotactic, phagocytic, and inflammatory pathways. Despite these advances, significant gaps remain in understanding the mechanistic basis of cell-mediated immunity in invertebrates and teleosts. Although these taxa possess broad immune gene repertoires, their functional roles are only partially characterized.

Delisle et al. report a major advance in oyster immunology by demonstrating that inactivated Ostreid herpesvirus-1 (OsHV-1) can trigger an innate immune response in the hemocytes of Pacific oyster. Using an innovative *in vitro* assay with hemocytes from *Crassostrea gigas* juveniles that are susceptible to OsHV-1, the authors tested multiple antigen preparations, including chemically/physically inactivated OsHV-1, viral DNA, and protein extracts. Ten different antigen preparations successfully stimulated hemocyte immune activity, without cytotoxic effects, benchmarked against the standard immune stimulant Poly (I:C). These findings represent a significant step toward developing of pseudo-vaccines for oysters, offering new strategies for managing polymicrobial diseases in aquaculture, such as oyster mortality syndrome (POMS), which has severely impacted global oyster aquaculture.

Emerging research on echinoderms reveals an astonishing immune complexity, reshaping the understanding of their immune cells and the molecular mechanisms underlying their defense strategies. A striking example comes from the sea cucumber *Holothuria forskali*. In their study, Wambreuse et al. overturned the long-standing belief that reddish coelomocytes were hemoglobin-containing respiratory cells. Through integrative morphological and multi-omic analyses, the authors demonstrate that these cells accumulate high levels of carotenoids, potent antioxidant compounds that confer their pigmentation. Based on these findings, the cells were redefined as “carotenocytes,” reflecting their role as specialized immune effectors that form antioxidant shells around pathogen-encapsulating aggregates to mitigate oxidative stress. This discovery represents a paradigm shift in understanding coelomocyte function and highlights the evolutionary complexity of echinoderm immunity.

The California purple sea urchin *Strongylocentrotus purpuratus* provides another compelling example of sophisticated innate immunity. Sea urchin defenses rely on the high variability of its SpTransformer (SpTrf) protein family, where different isoforms exhibit distinct capabilities in binding pathogens and enhancing phagocytosis. Crow et al. explored the role of six recombinant SpTrf proteins as opsonins in *S. purpuratus*. Their work demonstrates that different recombinant SpTrf proteins enhance immune activity in distinct phagocyte subtypes. When exposed to beads coated with different rSpTrf isoforms, polygonal phagocytes selectively bind and engulf a subset of rSpTrf::beads, discoidal cells maintain baseline phagocytosis for all rSpTrf::beads, while coelomocytes, previously inferred to have immune roles due to their increased numbers in response to immune challenges, are confirmed as phagocytes through their ability to engulf rSpTrf::beads. These findings underscore a functional specialization that contributes to a complex and effective invertebrate system for neutralizing a wide spectrum of pathogens.

To understand how functional specialization has developed in a different evolutionary lineage that possesses both innate and adaptive immunity, we now turn our focus to fish. Unlike the conventional view of red blood cells (RBCs) as oxygen carriers, research on teleost fish shows that they are actively involved in the immune response. Contrary to anucleate mammalian erythrocytes, fish RBCs retain their nucleus, enabling transcription and protein synthesis essential for immune functions. A study by Majstokovic et al. on the common carp *Cyprinus carpio* provides compelling evidence for the multifaceted immune role of RBCs. Through *in vitro* exposure to *Aeromonas hydrophila* and *in vivo* infections, the authors demonstrate that RBCs express pattern recognition receptors, including Toll-like receptors (TLRs), which enable pathogen detection, secrete pro-inflammatory cytokines, produce reactive oxygen species, and exhibit phagocytic activity. These findings position fish RBCs as immune sentinels, advancing our understanding of conserved immune mechanisms and their evolutionary significance in teleosts. Such insights have remarkable implications for aquaculture health management and the development of novel immunostimulatory strategies.

Complementing these findings, Dezfuli et al. explore the intricate co-evolutionary dynamics between fish and their parasites, describing an “arms race” in which helminths develop potent immune evasion strategies while fish evolve more sophisticated innate defenses. Their review details the critical role of innate immune cells, including mucous cells, mast cells, neutrophils, and macrophages, in response to parasite infections. These cells not only eliminate pathogens, but also orchestrate tissue repair and inflammatory modulation. Such findings shed light on the complexity of host-parasite interactions and their implications for fish health management.

From coelomocytes in echinoderms to macrophages in teleosts, aquatic species deploy a wide array of immune cells to combat pathogens and maintain homeostasis. Research by La Paglia et al. on *Holothuria tubulosa* reveal the proteomic complexity of coelomic fluid, identifying key proteins involved in phagocytosis, cytoskeletal remodeling, and signaling. Notably, the discovery of primary cilium-like structures in invertebrate immune cells provides novel insights into ancient, evolutionarily conserved immune signaling mechanisms.

A recurring theme across taxa is the role of pattern recognition receptors (PRRs), particularly TLRs, in pathogen detection and immune activation. Ghani et al. provide a comprehensive review of TLRs, emphasizing their evolutionary divergence and categorization, 3D structure, ligand recognition, signaling pathways, and expression dynamics during development. The review also highlights structural and functional differences between the TLRs in aquatic organisms and their mammalian counterparts, underscoring their unique roles in pathogen detection and immune adaptation in aquatic environments. Understanding these mechanisms offers promising strategies for aquaculture disease management and the development of targeted immunostimulants.

Collectively, the contributions in this Research Topic challenge traditional views of innate immunity in aquatic organisms. From hemocytes in oysters to coelomocytes in echinoderms and RBCs in teleosts, these studies illuminate a dynamic and multifaceted immune landscape shaped by evolutionary forces and environmental challenges. Beyond advancing fundamental knowledge, they open new avenues for aquaculture, environmental monitoring, and comparative immunology.

